# Supplementary material for: Comparing ecosystem gaseous elemental mercury fluxes over a deciduous and coniferous forest
Source: Nat Commun. 2023 May 11;14:2722. doi: 10.1038/s41467-023-38225-x (PMC10175444; doi:10.1038/s41467-023-38225-x)
Supplement: Supplementary file 1 — Supplementary Information [file 41467_2023_38225_MOESM1_ESM.pdf]

Supporting Information

Comparing ecosystem gaseous elemental mercury fluxes over a deciduous and coniferous forest

Jun Zhou[1, 2], Silas Bollen[1], Eric Roy[1, 3], David Hollinger[4], Ting Wang[1], John T. Lee[5], Daniel Obrist[1,6,]*

1. Department of Environmental, Earth and Atmospheric Sciences, University of Massachusetts, Lowell, MA, USA
2. Key Laboratory of Soil Environment and Pollution Remediation, Institute of Soil Science, Chinese Academy of Sciences, Nanjing 210008, China
3. Department of Earth, Atmospheric and Planetary Sciences, Massachusetts Institute of Technology, Cambridge, MA, USA
4. USDA Forest Service, Northern Research Station, Durham, NH, USA
5. School of Forest Resources, University of Maine, Orono, ME, USA
6. University of California, Agriculture and Natural Resources, Davis, CA, USA.

Corresponding author: Daniel Obrist (dobrist@ucanr.edu)

Text S1. Performance of flux-gradient method to measure GEM fluxes

The flux-gradient method quantifies GEM fluxes by measurement of GEM concentrations at two heights above a canopy multiplied by the turbulent exchange coefficient. Detection of small GEM fluxes that exist over terrestrial ecosystems – often below 1 ng m^{-2} hr^{-1} – is challenging because concentration differences measured between the two inlet heights are very small, a challenge amplified over forests due to high atmospheric turbulence which erodes concentration gradients[1]. Figure S4A shows 30-minute resolution GEM concentration differences above the coniferous forest canopy (upper inlet at 28.2 m minus lower inlet at 23.2 m; note that negative values show deposition). High turbulence levels above this forest caused concentration differences to be very small with a median value of -0.0018 ng m^{-3}, a mean of -0.0022 ng m^{-3}, and an interquartile range (IQR) of -0.0022 ng m^{-3} to 0.0063 ng m^{-3}. The measured GEM concentration differences were about three to four times lower than those reported over lower-statured ecosystems such as grassland and a tundra[2, 3]. Measurements of individual GEM gradients were often below the detection limit of the GEM analyzer (about 0.05 ng m^{-3} based on 3 standard deviations of 5-minute resolution measurements). We estimate a median GEM flux detection limit of 31 ng m^{-2} hr^{-1} at 30-resolution based three standard deviations of paired fluxes using a daily-differencing method (see below). The discussion above shows a need for substantial time averaging of 30-min. flux data to be able to analyze data, e.g., by calculating monthly hourly means and medians (e.g., 60 flux data points) to delineate diel patterns and by daily time averaging (e.g., 48 flux data points) to show daily GEM fluxes.

Measurements also require stringent quality assurance and control steps such as frequent filter exchange and line contamination and leak testing. An additional step was frequent rotation of the two entire inlet sampling train (i.e., lines and valves) to test for presence of null gradients. Null gradients are systematic biases in concentrations between inlet lines and become evident when inlet rotation results in significant changes and even reversal of concentration differences. We previously presented such quality assurance and control data for the deciduous Harvard Forest in Obrist et al.[4]. Here we perform the same analysis for the coniferous forest at Howland Forest in Maine. Figure S4B shows cumulative values of measured GEM concentration differences with positive slopes indicating periods of emissions and negative slopes periods of deposition. Vertical green lines signify times when the two sampling inlets to measure GEM gradients were rotated for quality control purposes to test for presence of null gradients (i.e., line biases, see Method). The slopes of the cumulative graphs and statistical comparisons of gradient measurements one day prior to and after inlet rotation show that inlet rotation did not significantly change fluxes or reverse flux directions.

To assess the statistical significance of measured GEM concentration differences, a cumulative periodogram (squared magnitude of the discrete Fourier transform, or power spectrum) was calculated (Figure S10A) which shows concentration differences to be statistically different from a white noise signal (Bartlett white-noise test: $p < 0.01$). This result confirms that at high frequencies, measured gradients were not based on random chance and represented an underlying exchange process that led to stratification of GEM concentrations in the lower boundary layer where GEM gradients were measured. Concentration differences also showed strong autocorrelations (Figure S10B) over short lag times (e.g., minutes), which declined over several hours, and showed renewed peaks at subsequent daily lag times providing evidence of strong diel cycles in measured GEM concentration differences. Such autocorrelation patterns are visible and exceeding 95% confidence intervals for about two weeks, after which autocorrelations ceased.

In summary, frequency spectra and autocorrelations of measured GEM concentration differences showed underlying periodicity patterns, statistical differences from white noise signals. In addition, strong autocorrelations at short time lags and underlying diurnal patterns provide confidence that GEM concentration differences were highly structured in time and controlled by meteorological and physiological variables which imposed diel variability in ecosystem GEM fluxes.

Text S2. Verification of flux-gradient method for CO_2 in comparison to Eddy Covariance fluxes

The flux-gradient method was chosen to quantify ecosystem-level GEM exchange as it is operable with low instrument time resolution (e.g. 2.5 to 5 minutes for GEM). Previous flux-gradient measurements at Howland Forest also were successfully conducted for greenhouse gases[5, 6] and water vapour[7], and one study compared flux-gradient data with Eddy Covariance (EC) data [8] and showed good methods agreements. We here also directly compared carbon dioxide (CO_2) fluxes measured by flux-gradient method deployed above the coniferous forest in Maine from May 1, 2021 to May 16, 2022 with CO_2 fluxes measured by the EC technique, which is the most commonly used and preferred method to quantify trace gas fluxes when high time resolution detection is available (>1 Hz)[9]. CO_2 fluxes above the canopy using both the EC and flux-gradient method using the same implementation as for GEM described by Meredith et al.[10] showed best agreement between methods with zero displacement heights of 19 m. CO_2 fluxes calculated from the gradients reproduced diel cycles well and quantitatively agreed with EC fluxes (Figure S8). Daily CO_2 fluxes during the intercomparison period amounted to 0.89 μmol m^{-2} s^{-1} using the EC method while the flux-gradient method resulted in 0.82 μmol m^{-2} s^{-1}, suggesting slight underestimation of fluxes by 7.8% using the

flux-gradient method in comparison to the EC method. A similar comparison between flux-gradient CO_2 fluxes using the two methods was also conducted in the deciduous forest at Harvard Forest and previously published by Obrist et al.[4].

Text S3. Random error estimation and error propagation of GEM flux measurements

To quantify uncertainties in measured GEM fluxes, we conducted a random error analysis of GEM flux data at Howland Forest in Maine as described for other micrometeorological flux measurements by Hollinger and Richardson [11] and as conducted for our first flux record at the deciduous forest at Harvard Forest[4]. Tower methods generally do not allow for spatial replication, so time can be traded for space for error analysis using a "daily differencing" approach. In this approach, random errors are estimated based on variability of 30-minute time resolution fluxes taken on two successive days. Paired flux data of two days are formed only for equivalent environmental conditions for fluxes measured at the same time and under similar photosynthetic active radiation (within 75 µmol m^{-2} sec^{-1}), temperatures (within 3 °C), and wind speeds (within 1 m sec^{-1}). A histogram of measurement differences of all data pairs is shown in Figure S9A. Relative random errors are quantified using standard deviations of daily-differences GEM flux pairs versus the magnitude of fluxes (using flux bins of 5 ng m^{-2} hr^{-1}, Figure S9A). Slopes are fitted to separately for GEM fluxes below zero (i.e., deposition) and above zero (emissions), whereby slopes (Δstdev/Δmean) represent relative standard deviations for each bin of GEM flux measurements.

Subsequently, we assigned normal distribution function for each measured data point using its flux value as mean and the standard deviation calculated based on the regression line (i.e., intercept plus slope; Figure S9B). For each measurements, 500 random data points were assigned based on respective normal distribution. Finally, cumulative sums of all 500 data columns generated were calculated for all 30-minuted GEM flux observations and 95% confidence intervals were calculated. The original 15- and 18-months cumulative measurement records including random error estimations were calculated and shown in Figure S2. The uncertainty in a measurement can also be characterized by making multiple measurements of a process and then using the variability of these measurements to estimate the standard deviation of the uncertainty, which is detailed by Hollinger and Richardson[12]. Briefly, A pair of independent flux measurements made repeatedly under identical conditions (ideally, this would mean simultaneously), provides a solution to many of the problems described above. In this case, if the flux process has true value $\bar{x}$, we will actually measure the following pair X_1, X_2:

$X_1 = \bar{x} + \delta q_1$ (1)

$X_2 = \bar{x} + \delta q_2$ (2)

Here the measurement uncertainty (δq_i) is a realization of a random variable with mean 0 and standard deviation $\delta(\delta q)$. To characterize the uncertainty in our measurements, we want to determine the value of $\sigma(\delta q)$. Because the expected value of $(X_1 - X_2)$ is 0, the variance of $(X_1 - X_2)$ equals the variance of $(\delta q_1 - \delta q_2)$, which is given by:

$$\sigma^2(\delta q_1 - \delta q_2) = \sigma^2(\delta q_1) + \sigma^2(\delta q_2) + 2\mathrm{cov}(\delta q_1, \delta q_2) \quad (3)$$

Since δq_1 and δq_2 are independent and identically distributed, the right hand of Equation 3 simplifies to $2\sigma^2(\delta q)$. Thus:

$$\sigma(\delta q) = \frac{1}{\sqrt{2}}\sigma(X_1 - X_2)$$

Therefore, by repeating the paired measurements X_1 and X_2, we can estimate $\sigma(\delta q)$ by calculating the standard deviation of the difference $(X_1 - X_2)$.

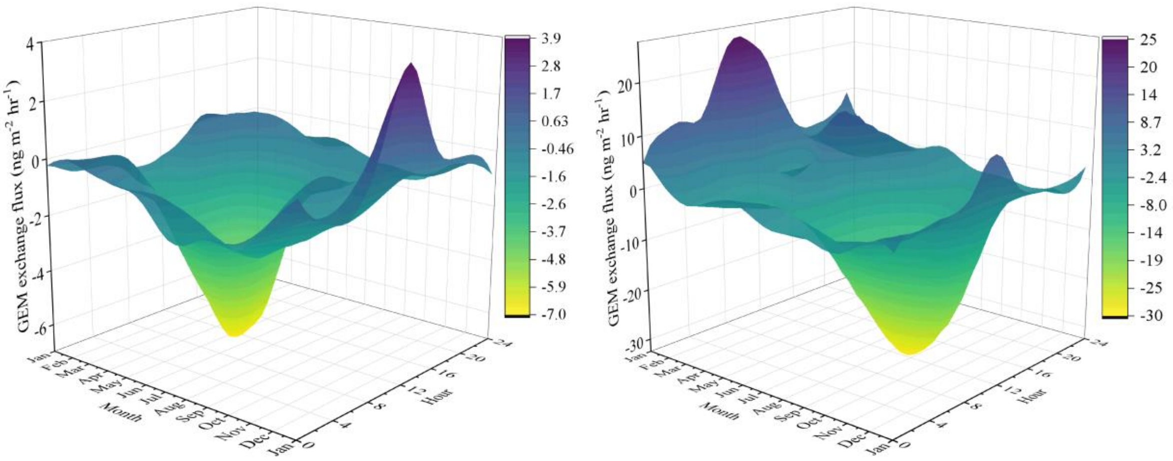

Figure S1. Whole ecosystem gaseous elemental mercury (GEM) fluxes. 3-D graphs of seasonal and diurnal variations of GEM fluxes measured in the coniferous forest in Maine, USA (left panels) and deciduous forest in Massachusetts (right panels). Negative GEM fluxes denote deposition and positive fluxes represent emissions.

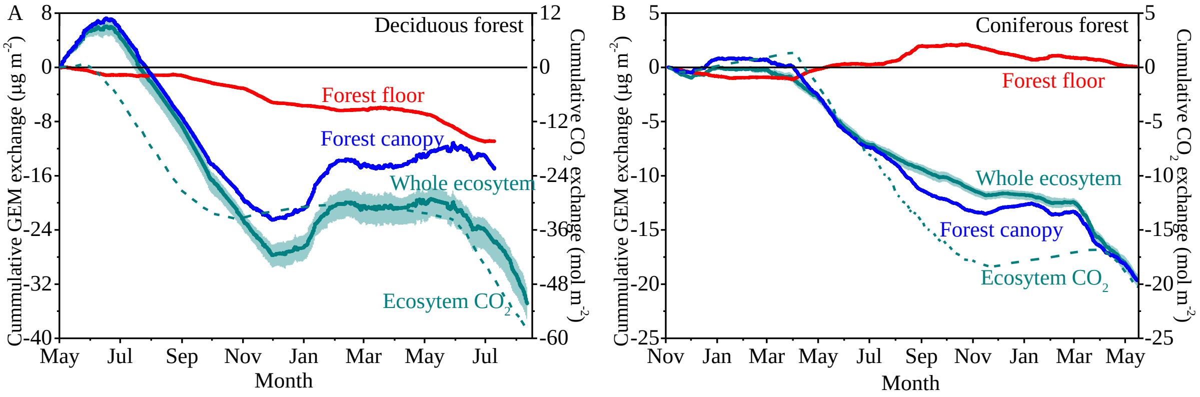

Figure S2. Cumulative gaseous elemental mercury (GEM) fluxes of whole-ecosystem, forest floor and forest canopy fluxes in the deciduous (A) and coniferous (B) forests during 15 months in the deciduous forest and 18 months in the coniferous forest.

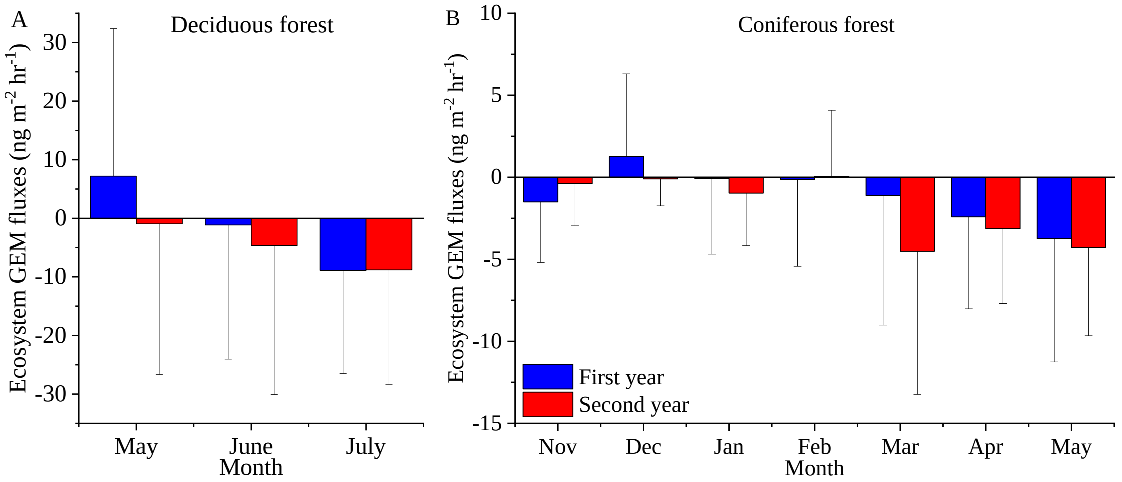

Figure S3. The whole ecosystem gaseous elemental mercury (GEM) fluxes of the repeat months observed in the deciduous forest (A) and coniferous forest (B). Bars represent standard deviation of half-an-hour fluxes (n > 1000).

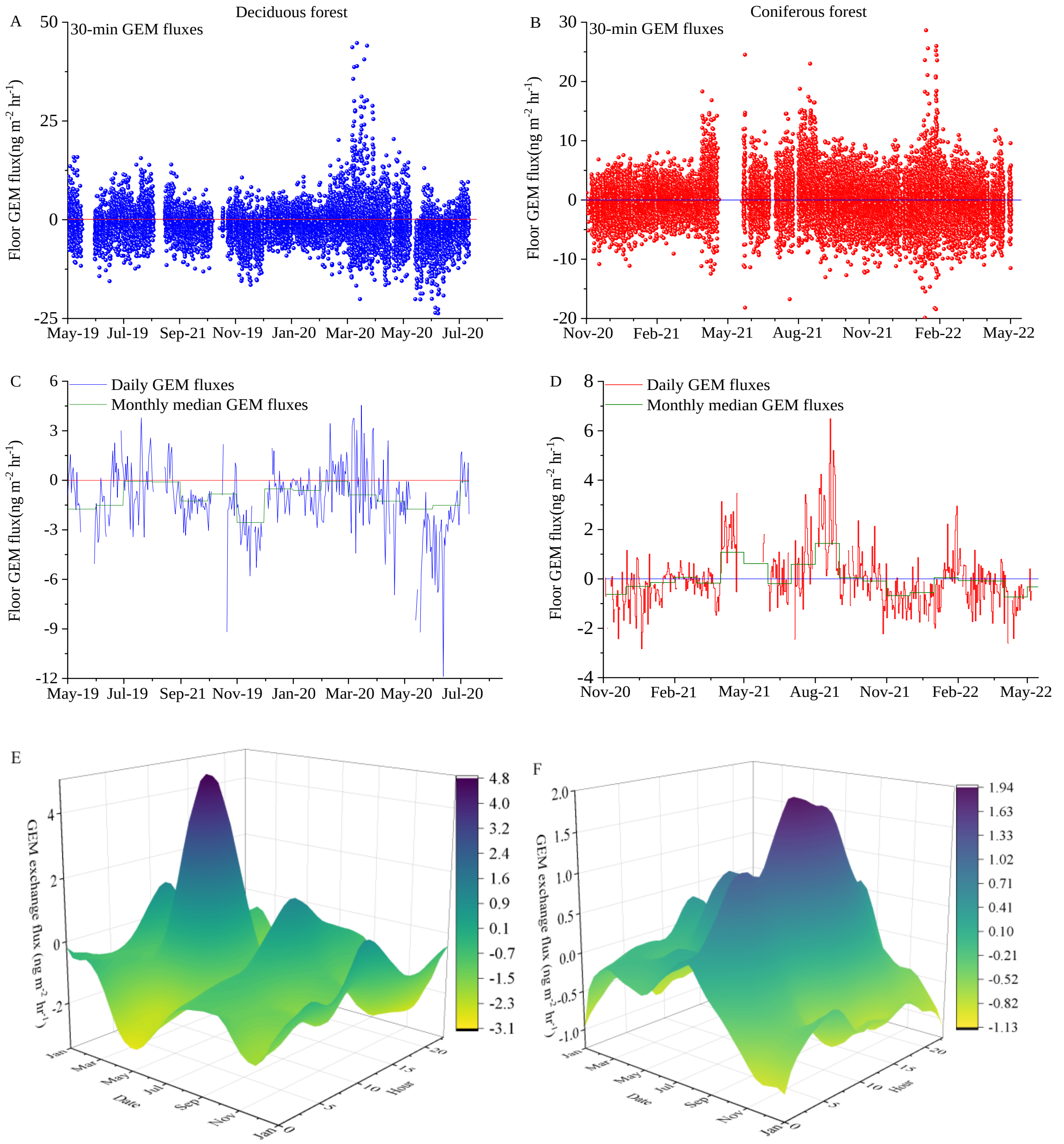

Figure S4. Forest floor gaseous elemental mercury (GEM) fluxes. 30-min resolution GEM exchange fluxes (panels A and B), daily mean GEM fluxes and median monthly GEM fluxes (C and D), and 3-D graphs of seasonal and diurnal variations (E and F) of GEM fluxes measured over the deciduous forest (left panels) and coniferous forest floor (right panels). Negative GEM fluxes denote deposition and positive fluxes represent emissions.

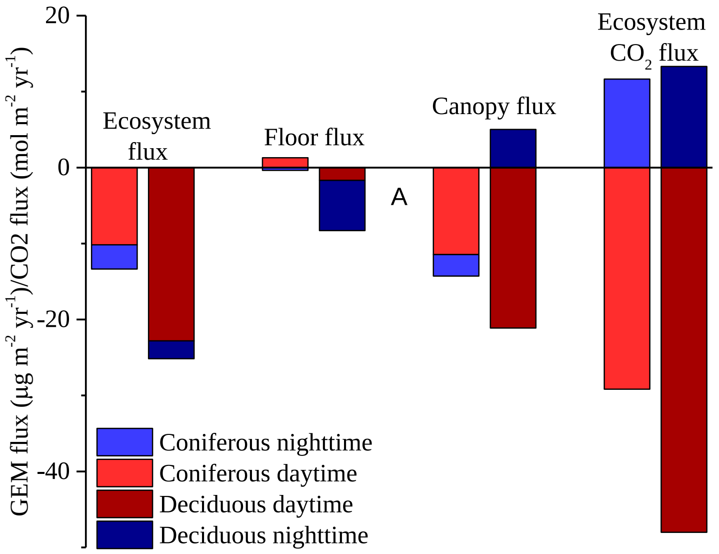

Figure S5. Bar graphs showing cumulative annual GEM and CO_2 fluxes for whole ecosystem, forest floors, and canopy (by difference) for the coniferous and deciduous forest. Fluxes are separated into nighttime fluxes (blue colors) and daytime fluxes (red colors).

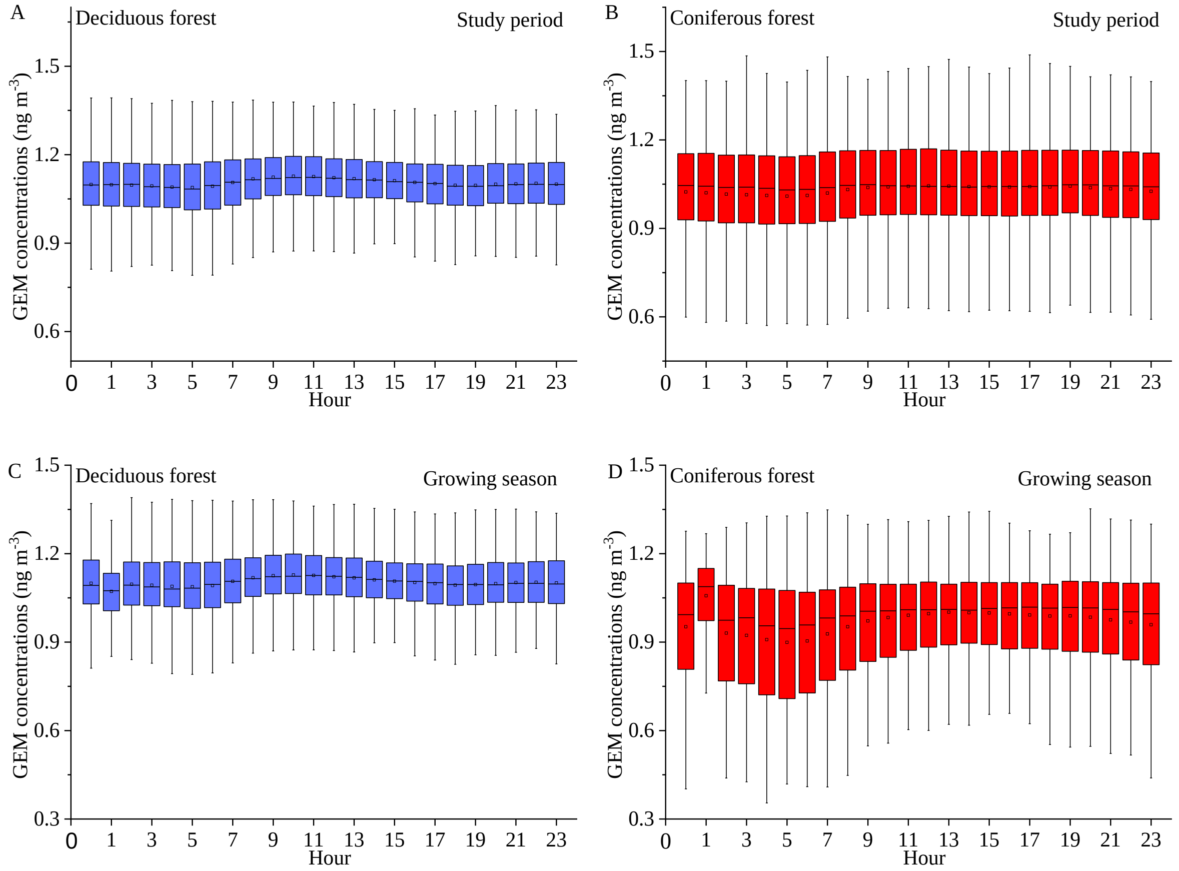

Figure S6. Diurnal variations of gaseous elemental mercury (GEM) concentrations in the deciduous forest (A and C) and coniferous forest (B and D).

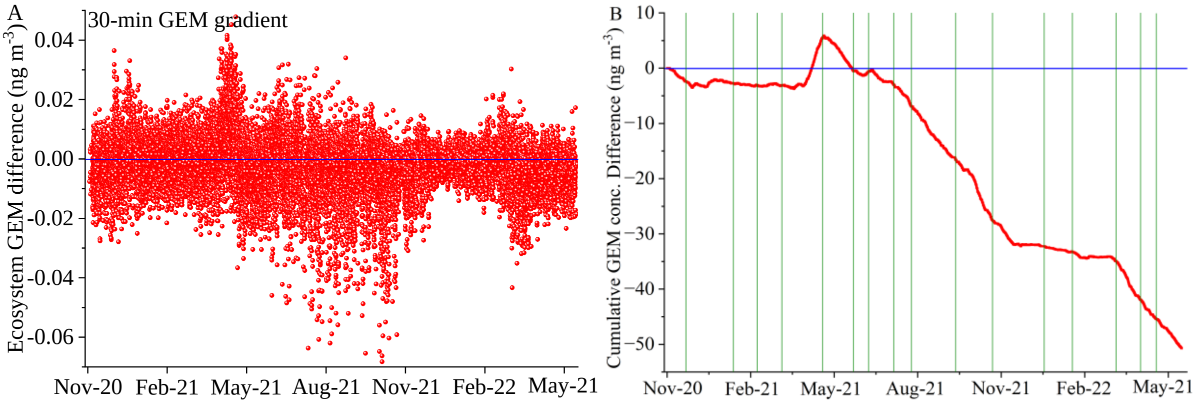

Figure S7. A. 30-minute resolution GEM concentration differences (lower inlet – upper inlet concentrations) above the forest canopy at Howland Forest. **B.** Cumulative sum of 30-minute resolution concentration differences with slope direction indicating direction of fluxes (positive slopes emission, negative slopes deposition). Note that vertical green lines show times when inlet slopes were rotated which did not reverse flux directions and showed not significant difference in slope between one day of data prior to and after the line switch.

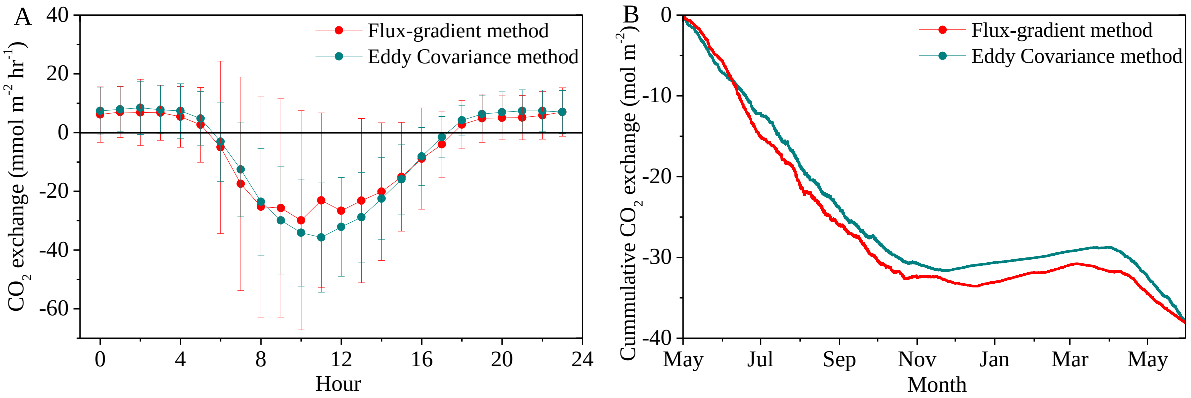

Figure S8. Comparison of ecosystem-level (i.e., above canopy) CO_2 fluxes measured by Eddy Covariance method and flux-gradient method following Edwards et al.[13] at Howland Forest from May 1, 2021 to May 15, 2022. Data points represent hourly averaged data for the growing seasons (April- October) (A) and cumulative annual CO_2 fluxes measured for the full time period (B). Bars represent standard deviation of hourly replicates in the growing season ($n > 330$).

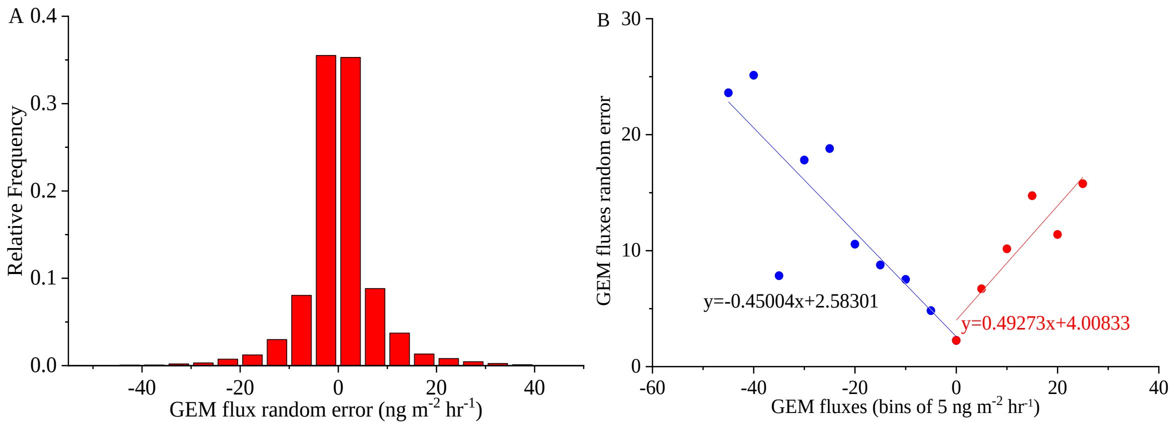

Figure S9. A. Error propagation of gaseous elemental mercury (GEM) fluxes using "daily difference" approach. Histogram of "daily difference" pairs of GEM observations taken at the same time on two successive days under equivalent environmental conditions. B. Relative random errors quantification using scatter plots of mean standard deviations versus flux magnitude (using flux bins of 10 ng m^{-2} hr^{-1}). Regression lines stdev/mean are a measure of relative standard deviation for each bin of GEM flux measurements.

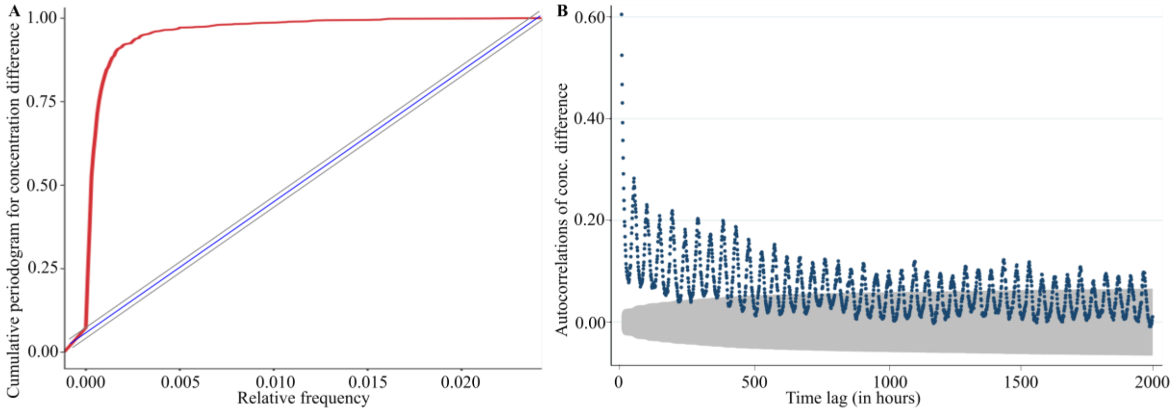

Figure S10. A. Cumulative spectral distribution analyses of measured 30-minute gaseous elemental mercury (GEM) concentration differences above the forest canopy. X-axis represent relative frequency based on 22,536 data points. The spectral distribution of a random signal is represented by the three linear gray lines (mean and 95% confidence intervals of a white noise signal). Bartlett statistics shows measurements to be statistically different from a white noise signal ($p < 0.01$). B. Autocorrelation (y-axis) of measured GEM concentration differences against time lag of data (x-axis). Strong autocorrelation are visible over short time scales with local peaks in autocorrelations at daily lag intervals. Shaded area represents 95% confidence interval based on Bartlett's formula.

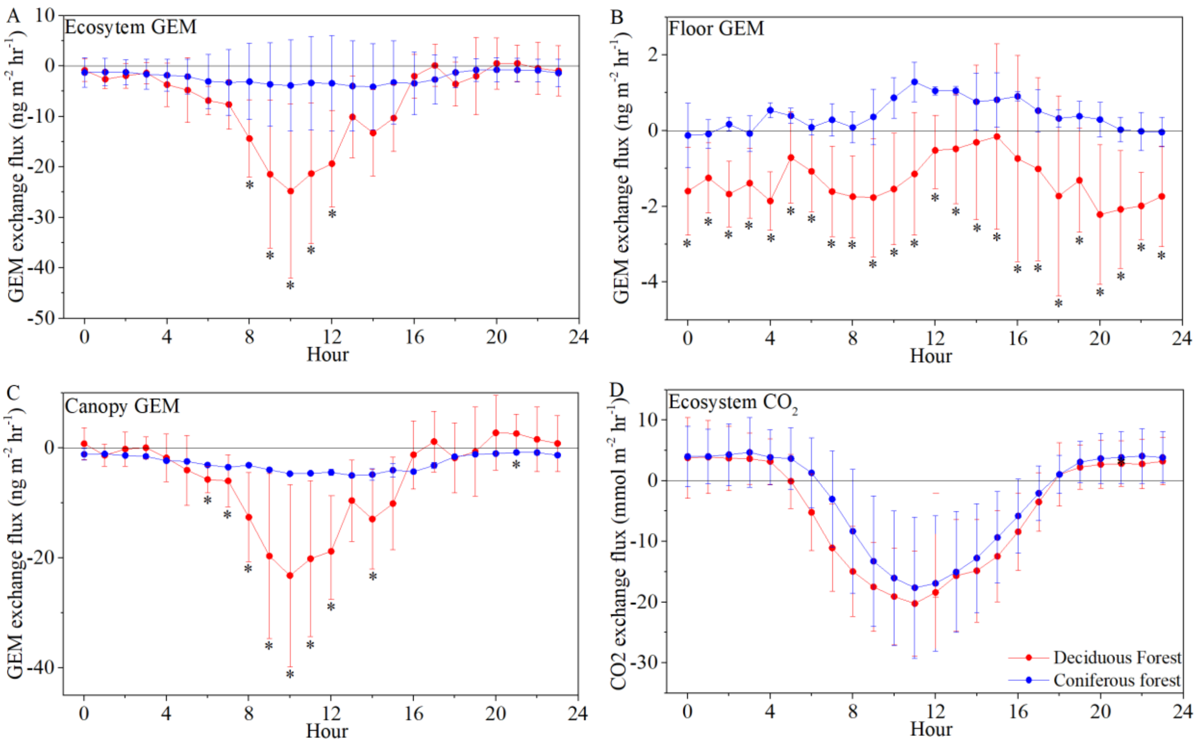

Figure S11. Diel patterns of gaseous elemental mercury (GEM) fluxes for ecosystem-level (A), forest floors (B), canopy (C), and ecosystem CO_2 (D) fluxes for the deciduous forest and coniferous forest Bars represent standard deviation of hourly replicates (n > 90).* represent statistically significant ecosystem GEM fluxes for each hour at the $p < 0.05$ level.

References:

1. Agnan Y, Le Dantec T, Moore CW, Edwards GC, Obrist D. New constraints on terrestrial surface atmosphere fluxes of gaseous elemental mercury using a global database. *Environmental Science & Technology* **50**, 507-524 (2016).

2. Fritsche J, Obrist D, Zeeman MJ, Conen F, Eugster W, Alewell C. Elemental mercury fluxes over a sub-alpine grassland determined with two micrometeorological methods. *Atmospheric Environment* **42**, 2922-2933 (2008).

3. Obrist D, *et al.* Tundra uptake of atmospheric elemental mercury drives Arctic mercury pollution. *Nature* **547**, 201-204 (2017).

4. Obrist D, *et al.* Previously unaccounted atmospheric mercury deposition in a midlatitude deciduous forest. *Proceedings of the National Academy of Sciences of the United States of America* **118**, e2105477118 (2021).

5. Marino BD, Bautista N, Rousseaux B. Howland Forest, ME, USA: Multi-gas flux (CO2, CH4, N2O) social cost product underscores limited carbon proxies. *Land* **10**, 436 (2021).

6. Richardson AD, Hollinger DY, Shoemaker JK, Hughes H, Savage K, Davidson EA. Six years of ecosystem-atmosphere greenhouse gas fluxes measured in a sub-boreal forest. *Scientific Data* **6**, 117 (2019).

7. Hollinger D, Goltz S, Davidson E, Lee J, Tu K, Valentine H. Seasonal patterns and environmental control of carbon dioxide and water vapour exchange in an ecotonal boreal forest. *Global Change Biology* **5**, 891-902 (1999).

8. Wehr R, Saleska SR. Calculating canopy stomatal conductance from eddy covariance measurements, in light of the energy budget closure problem. *Biogeosciences* **18**, 13-24 (2021).

9. Muller JBA, Coyle M, Fowler D, Gallagher MW, Nemitz EG, Percival CJ. Comparison of ozone fluxes over grassland by gradient and eddy covariance technique. *Atmospheric Science Letters* **10**, 164-169 (2009).

10. Meredith LK, *et al.* Ecosystem fluxes of hydrogen: a comparison of flux-gradient methods. *Atmospheric Measurement Techniques* **7**, 2787-2805 (2014).

11. Hollinger D, Richardson A. Uncertainty in eddy covariance measurements and its application to physiological models. *Tree physiology* **25**, 873-885 (2005).

12. Hollinger DY, Richardson AD. Uncertainty in eddy covariance measurements and its application to physiological models. *Tree Physiology* **25**, 873-885 (2005).

13. Edwards G, *et al*. Development and evaluation of a sampling system to determine gaseous Mercury fluxes using an aerodynamic micrometeorological gradient method. *Journal of Geophysical Research: Atmospheres* **110**, D10306 (2005).
