# Supplementary material for: Comparing ecosystem gaseous elemental mercury fluxes over a deciduous and coniferous forest
Source: Nat Commun. 2023 May 11;14:2722. doi: 10.1038/s41467-023-38225-x (PMC10175444; doi:10.1038/s41467-023-38225-x)
Supplement: Supplementary file 3 — Description of Additional Supplementary Files [file 41467_2023_38225_MOESM3_ESM.pdf]

Description of Additional Supplementary Files:

Supplementary Data 1: Gaseous elemental mercury flux in coniferous forest.
